# Autoscopic Hallucination in Alcohol Dependence Syndrome: A Rare or Missed Phenomenon?

**DOI:** 10.1155/2017/2598973

**Published:** 2017-07-24

**Authors:** Sulochana Joshi, Binita Thapa, Rabi Shakya

**Affiliations:** Department of Psychiatry, Patan Academy of Health Sciences, Lalitpur, Nepal

## Abstract

Autoscopic phenomenon, a psychic illusionary duplication of one's own self, has been the subject of interest in the literature and science for years. It has been reported in various diseases of the central nervous system but with an unknown mechanism. Hallucinations are a common presentation in alcohol dependence syndrome during delirium tremens and as induced disorder. However, autoscopic hallucination has been rarely reported in the cases of alcohol dependence. We present a case of a 40-year-old man who experienced autoscopic hallucination during the withdrawal state of alcohol. He was successfully treated with detoxification and an antipsychotic medication and was doing well. The case highlights the need for strong suspicion and exploration of the autoscopic hallucination and autoscopic phenomenon in general in cases of alcohol dependence syndrome.

## 1. Introduction

Autoscopy means seeing own self, derived from the Greek words “autos” (self) and “skopeo” (looking at) [1]. Autoscopy is defined as a visual experience where the subject sees an image of him/herself in external space, viewed from within his/her own physical body [2]. Autoscopic phenomena are psychic illusory visual experiences consisting of the perception of the image of one's own body or face within space, either from an internal point of view, as in a mirror, or from an external point of view [1]. There are 3 main forms of this phenomena described on the basis of phenomenological and anatomical criteria, that is, heautoscopy, autoscopic hallucination, and out-of-body experience [3]. In an autoscopic hallucination the observer's perspective is clearly body centered, and the visual image of one's own body appears as a mirror reversal [4].

The phenomena have been reported in various diseases of central nervous system like meningitis and encephalitis, intoxications, generalized epilepsies [5], focal epilepsy [6], space occupying lesions [7], and dysembryoplastic neuroepithelial tumor [6]. Other causes include migraine and psychiatric disorders, pharmacological agents, and altered psychological states [1, 8]. Autoscopic hallucinations are observed in infectious diseases especially typhus, brain tumors, and posttraumatic cerebral lesion [9].

Generation of autoscopic phenomena involves integration of many senses; however, the central mechanism is still obscure. Despite being of literary interest since years, the cases of autoscopic phenomena are rare and data is scarce in context of alcohol dependence syndrome.

In the present case report, we discuss autoscopic hallucination in a patient of alcohol dependence syndrome who presented with hallucinatory phenomena during withdrawal state.

## 2. Case

A 40-year-old social worker educated up to intermediate level with past history of hypertension under medication amlodipine 5 mg for the last 8 years was admitted in psychiatry ward for fearfulness for 1 day from out-patient department. On further elaboration, it was revealed that he used to drink alcohol for the last 20 years, the amount and frequency of which had gradually increased over time and at times drinking to the point of losing control over oneself. He also had strong desire or sense of compulsion to take it and neglect alternative pleasures or interests because of its use. His last alcohol intake was few days prior to admission; otherwise he had never quit drinking alcohol before this. Also, the family noticed change in his behaviour which was acute in onset characterized by suspiciousness towards his wife and surrounding expressing that the policemen and other people carrying guns are following him and conspiring to kill him. He further explained that he could not trust anyone as he was firm on his belief of being harmed. Since then he had decreased sleep and appetite. He also reported seeing his own image which followed and imitated him but did not communicate with him. He described seeing the image couple of times during dawn and dusk time for about 2-3 seconds expressing the image to be his exact double as if seeing oneself in the mirror in amazement. The image was mirror reversed, equally prominent on both sides of visual space without any elaboration of the other aspects of the visual space. Further, he elaborated seeing other people around him in small sizes in the initial few days. Patient was apprehensive and guarded. His predominant mood was fearful. On examination patient was restless, vigilant, and denied of craving with motivation in contemplation phase. He was fearful and had delusion of persecution and Autoscopic and Lilliput hallucination saying he had never experienced these before.

On physical examination, he was averagely built with minimal tremors of hand on extension. His vitals were stable. No abnormalities were detected in systemic examination. Baseline investigations, that is, complete blood count, liver function test, kidney function test, serum electrolytes, chest X-ray (P/A view), and ECG, were within normal limits; however, liver function test was slightly deranged and USG abdomen revealed hepatomegaly with fatty changes. EEG, though planned, could not be done as it was not available in the hospital and refused by the patient and the attendant. An ICD diagnosis of mental and behavioural disorders due to use of alcohol in withdrawal state in delirium was made.

He was detoxified with tablet Lorazepam 10 mg/day along with multivitamin thiamine 300 mg/day in divided doses. Also, tab risperidone 2 mg/day was added for his symptoms. Within four days of admission patient improved gradually and there were no behavioural symptoms. The Lilliputian hallucination was gone after the second day of admission and the autoscopic hallucination remained till the fifth day as inpatient. However, he still harboured persecutory ideas but not apprehensive as before. Ophthalmology consultation revealed Presbyopia. Motivation regarding abstinence and relapse prevention was continued during the hospital stay. The benzodiazepine was tapered off by 25 percent on sixth day. Patient was then discharged on 8th day of admission after reaching the premorbid state and was free from any persecutory thoughts.

In 1 week follow-up, he was doing well and not drinking alcohol. Lorazepam dose was further decreased and ultimately stopped. The plan to taper off risperidone was made. The patient was doing well in the second follow-up (after 2 weeks of first follow-up) with motivation in decision phase and without any behavioural symptoms. Risperidone was decreased to 1 mg and it was planned to stop in the next visit. Rigorous sessions on motivation enhancement and relapse prevention were continued in every follow-up.

## 3. Discussion

This case report documents the presence of autoscopic hallucination in the alcohol withdrawal state, which is an interesting finding. In this phenomenon, the index case saw himself for around 3 seconds, imitating himself during dawn and dusk time period in clear consciousness along with another psychopathology. Visual hallucinations as one of the psychotic symptoms are quite common in conditions related to alcohol like intoxication, withdrawal, alcohol-induced psychotic disorder, and delirium tremens; however, in such cases one sees insects crawling, animals, and other people.

In this case report, we observed autoscopic hallucination which is quite rare in a clinical setting. This autoscopic phenomena are not completely understood. It is hypothesized that a failure to integrate multisensory signals at the temporoparietal junction, resulting in a breakdown of the spatial unity between self and the body, causes autoscopy. The contribution of visual and somatosensory cues of self-location is largely from clinical and experimental data and little is known about the role of vestibular cues. Experimental data suggest that autoscopic hallucination is associated with damage to the right parietooccipital cortex [1]. The index case shows that there could be some causal relationship between autoscopic hallucination and alcohol dependence. The rarity could be explained on the grounds of inadequate exploration of alcohol dependence syndrome patients for the phenomena of autoscopy and more emphasis being given on the acute management and relapse prevention. Another reason could be the duration of phenomena which is transient making it difficult to pick and attribute.

To conclude this case highlights the need of meticulous observation and exploration of the autoscopic phenomenon in the cases of alcohol dependence syndrome for the better understanding of the phenomena and the association with the dependence syndrome.
